# Methylation of S100A8 is a promising diagnosis and prognostic marker in hepatocellular carcinoma

**DOI:** 10.18632/oncotarget.10792

**Published:** 2016-07-23

**Authors:** Kun Liu, Yuening Zhang, Chengdong Zhang, Qinle Zhang, Jiatong Li, Feifan Xiao, Yingfang Li, Ruoheng Zhang, Dongwei Dou, Jiezhen Liang, Jian Qin, Zhidi Lin, Dong Zhao, Min Jiang, Zhenxin Liang, Jie Su, Vanaparthy Pranay Gupta, Min He, Xiaoli Yang

**Affiliations:** ^1^ Medical Scientific Research Center, Guangxi Medical University, Nanning, Guangxi 530021, China; ^2^ Genetic and Metabolic Central Laboratory, The Maternal and Children Health Hospital of Guangxi, Guangxi 530002, China; ^3^ Department of Endocrinology, Fourth Affiliated Hospital of Guangxi Medical University, Liuzhou, Guangxi, 545005, China; ^4^ Department of Otolaryngology-Head and Neck Surgery, First Affiliated Hospital of Guangxi Medical University, Guangxi 530021, China; ^5^ Department of Gastroenterology, The Third Hospital of Nanchang City, Jiangxi 330009, China; ^6^ Department of Microbiology and Microbial Engineering, School of Life Sciences, Fudan University, Shanghai 200438, China; ^7^ School of Public Health, Guangxi Medical University, Guangxi 530021, China; ^8^ Key Laboratory of Early Prevention and Treatment for Regional High Frequency, Ministry of Education, Guangxi Medical University, Nanning, Guangxi 530021, China

**Keywords:** hepatocellular carcinoma, S100A8, DNA methylation, pyrosequencing, prognosis

## Abstract

The abnormality of DNA methylation is one of the major epigenetic alterations in the human hepatocellular carcinoma (HCC). We have assessed the global genomic DNA methylation profiles in human HCC patients by using the Infinium Human Methylation27 BeadChip. A CpG loci of S100A8 was found to be significantly hypomethylated in HCC.

Pooled meta-analysis of five validation public datasets demonstrated its methylation level was significantly lower for HCC compared to paired adjacent normal tissues. Quantitative pyrosequencing analysis also showed that the S100A8 methylation level was decreased in cancer tissues (31.90%±13.31%) than that in the paired adjacent normal tissues (65.33%±3.64%, *p*<0.01). The area under the ROC curve (AUC) value was 0.950 (*p*<0.01). Kaplan-Meier survival curves revealed that hypomethylation of S100A8 was associated with shortened overall survival (OS) and progression-free survival (PFS) (log rank *p*<0.05). Multivariate Cox proportional hazards model also indicated significantly shorter OS (HR, 1.709; 95 % CI, 1.127–2.591) and PFS (HR, 1.767; 95 % CI, 1.168–2.974) were observed in the low-methylation-level group compared to the high-methylation-level group. Furthermore, S100A8 overexpression in Huh7 and MHCC-97H hepatoma cell lines led to increased cell proliferation, migration, invasion, and tumor growth. These findings suggested S100A8 methylation to be served as potential diagnosis and prognosis marker for HCC. S100A8 also may play as a tumor promoter in HCC.

## INTRODUCTION

Hepatocellular carcinoma (HCC) is one of the most common and aggressive tumors. Not only its incidence expands in the past decades, HCC is also a leading cause of cancer-related deaths worldwide [1–4]. Although considerable efforts that have been made in the treatment of HCC such as surgical resection, liver transplantation and chemotherapy, the mortality rate remains high, and it is largely due to relapse after surgery or formation of intra-hepatic metastases [5–7]. Given the fact that most HCC patients at the advanced stage are prone to present a poor prognosis, there is an urge for the identification of effective diagnostic and prognostic biomarkers for HCC. Currently, the serum AFP test is widely used, however, with a relatively low sensitivity, thus its application is barely satisfactory and its clinical value is limited [8].

Aberrant DNA methylation is a common event during the pathogenesis of human cancers and one of the important epigenetic mechanisms in carcinogenesis. Loss of methylation primarily affects repetitive genomic elements and gene bodies, while hypermethylation mostly occurs at the promoters of tumor suppressor genes [9–12]. Many studies have provided evidence that cancer-linked DNA methylation alterations may be used as early indicators of HCC, as well as prognostic markers of cancer progression and response to chemotherapy [13, 14]. Until recently, mounting evidence indicates that the hypomethylation of “normally” methylated genes are significant in the pathogenesis of HCC. A number of hypomethylated tumor-promoting genes, including LINE-1, DNC, HPA, TFF3, MAT2A, HKII, CD147 and VIM have been identified in primary human HCC [15–18].

S100A8, also known as calcyclin, is a low-molecular-weight calcium-binding protein which belongs to the S100 family. S100A8 is involved in the regulation of a wide range of cellular processes, such as cell proliferation, the dynamics of cytoskeleton components, differentiation, Ca^2+^ homeostasis, and apoptosis [19, 20]. The expression of S100A8 is in a cell-specific manner, mainly in fibroblasts and epithelial cells [21, 22]. Furthermore, high levels of S100A8 have been found in some diseases, especially in cancer. So far the protein level of S100A8 has been studied by immunohistochemistry and shown to be up-regulated in colorectal carcinoma, lung cancer, pancreatic cancer, breast cancer and thyroid carcinoma [23–30]. Expression of several members of the S100 family, including S100A2, S100A4, S100A6, and S100P, are known to be regulated epigenetically. The declining expression of S100A8/A9 in HNSCC patients is associated with increased DNA methylation [31]. DNA demethylation resulted in S100A8 increased in myeloid cells [32]. There was rare study of S100A8 methylation in HCC.

In the present study, we analyzed the paired samples of tissues from the HCC patients to find a novel methylation alteration through genome-wide DNA methylation array screening and meta-analysis. After that, pyrosequencing was used to quantify the methylation levels of S100A8 in HCC samples, and the diagnostic value of S100A8 was examined. We further explored the potential role of S100A8 methylation in HCC prognosis using TCGA microarray datasets. At last, the function of S100A8 in HCC cell lines were investigated.

## RESULTS

### Methylation microarrays

**Figure 1:**
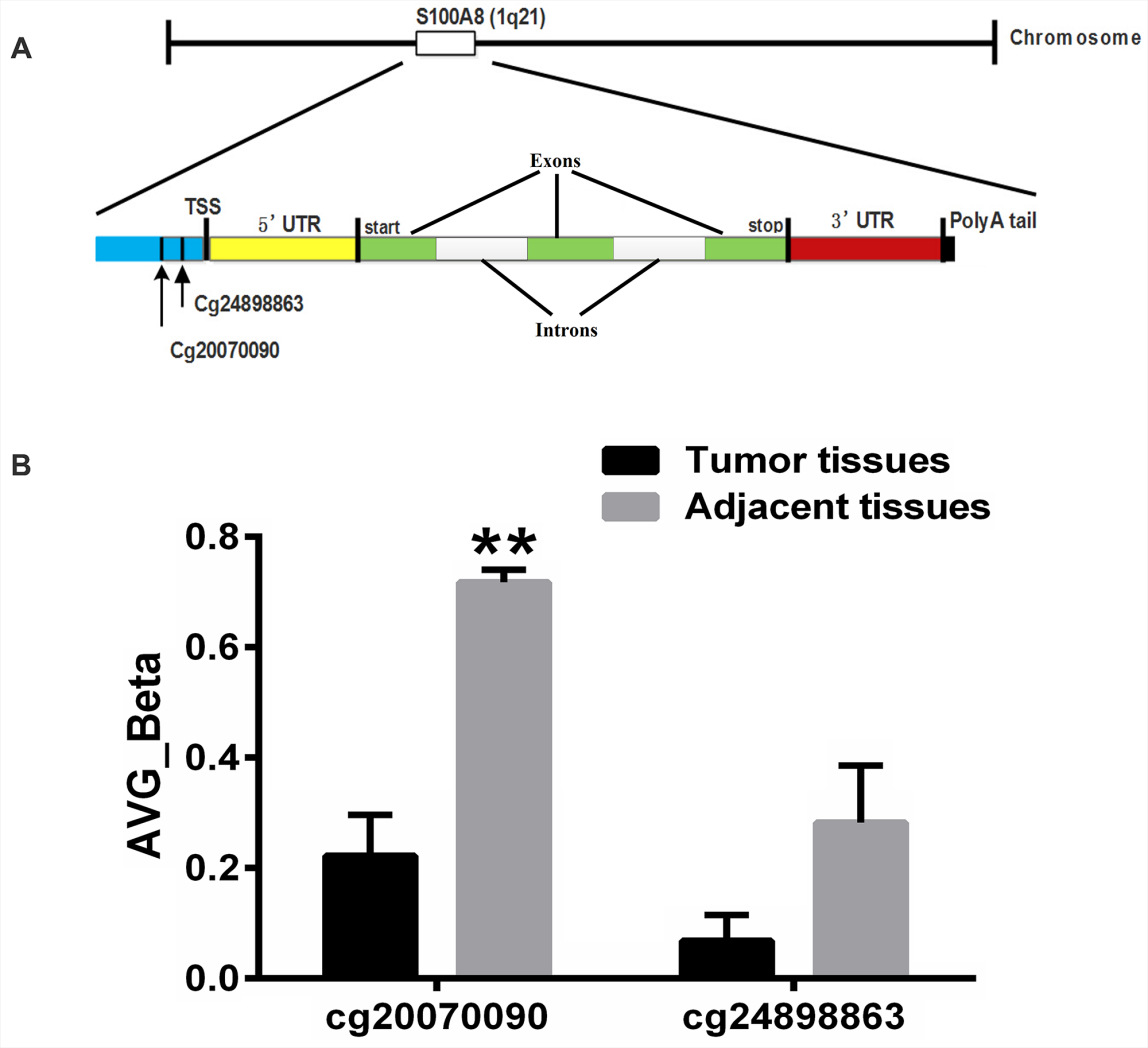
Gene structure of S100A8 **A.** S100A8 gene is located in the 1q21 chromosome. S100A8 consists of three exons and two introns. The two methylation sites of the gene are upstream of transcript start site. **B.** Average Beta of methylation sites in S100A8. Test sites are listed on the x axis, and the mean methylation level is shown on the y axis. Significant differences (*p*<0.01) are denoted with two asterisks.

Global DNA methylation profiles were measured by Illumina Infinium Human Methylation27 BeadChips, which target 14,475 total refseq genes, 12,833 well-annotated genes described in the NCBI CCDS database, 144 methylation hotspots in cancer genes, 982 cancer-related targets and 110 miRNA promoters. We performed genome-wide methylation profiling with 3 HCC tissues and their adjacent normal tissues. Two methylation sites of S100A8 were detected (Figure 1A and Table 1). As shown in Figure 1B, the CpG site (cg20070090) of S100A8 is hypomethylated in three pairs of tissues (*p*<0.01), while the CpG site (cg24898863) is not changed significantly.

**Table 1:** Sequence information of two methylation sites of the S100A8

Target ID	Symbol	Sequence	CpG island	Distance to TSS
cg20070090	S100A8	TCTCCTCTCTCAGGAA GGCTGCTCCACTTCCCTG ACCCTCCCCAAGAGAAGCCC AAAGTG[CG]GGGCCA ACCCAGACAGTCCCA CTTACCAGGTCTTCTGAAA GACAGCTGACAAGAGACATG	FALSE	60
cg24898863	S100A8	GTCTTCTGAAAGACAG CTGACAAGAGACATGCAGG GCTGAGAGGCAGCTC CTTTTTATAG[CG]GTTA GGCTTGGCCAGCTGCC CACAGCTTCAG GCCATCAGAGACAGC TTCTCCCTGCCAGA	FALSE	31

### Meta-analysis of five HCC methylation expression datasets identifies hypomethylation of S100A8

**Figure 2:**
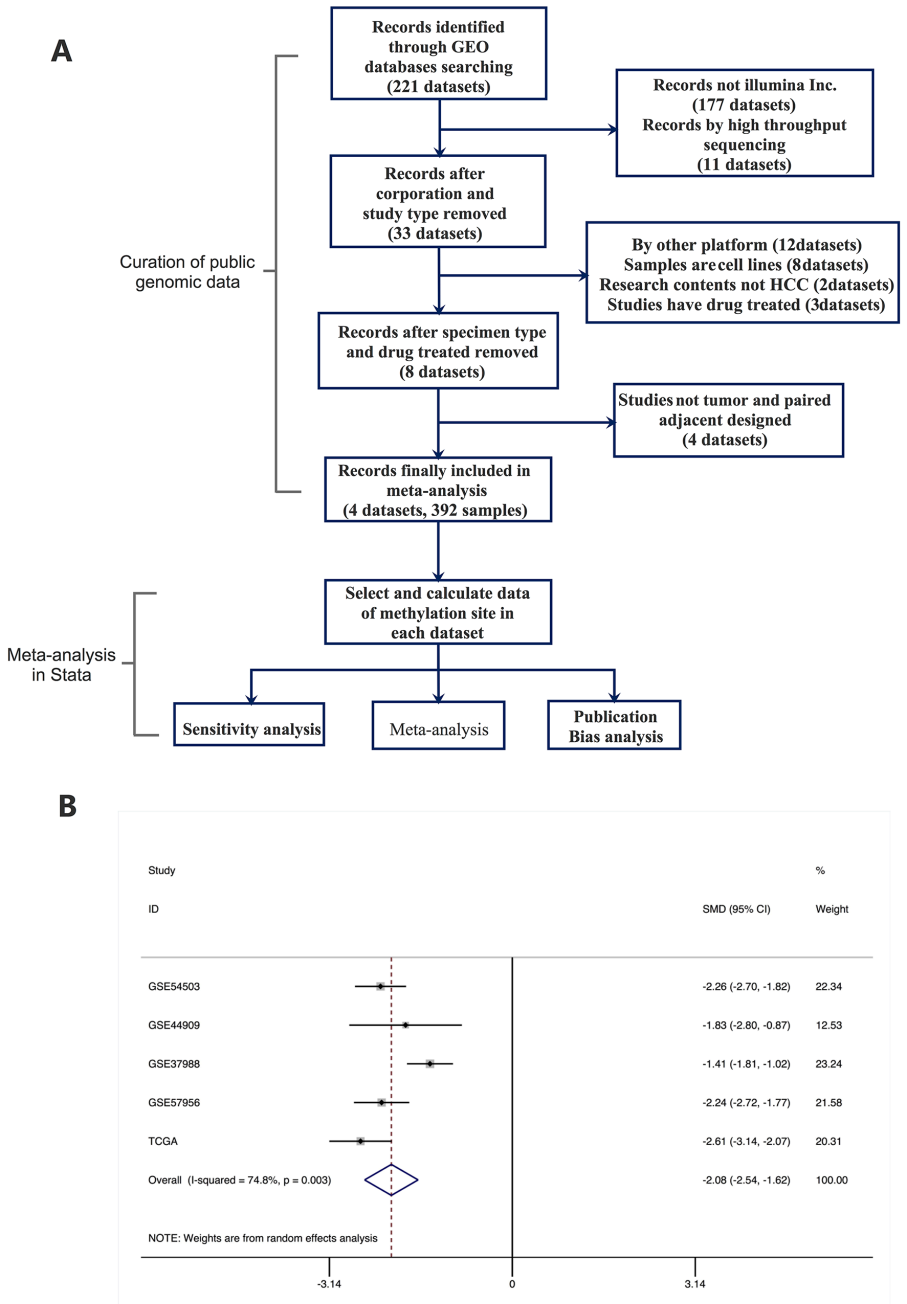
Meta-analysis of methylation cite of S100A8 **A.** Flow chart of Meta-analysis. **B.** Forest plot for the association between S100A8 Methylation and HCC using a random-effect model. SMD: standard mean difference (tumor vs. adjacent normal tissue).

We identified and manually curated five published and publicly available HCC datasets. Only datasets containing both HCC and their adjacent normal samples were used for further analysis. Four datasets from GEO and part of the TCGA dataset finally met this criteria (246 HCC, 246 adjacent normal; Supplementary Table S1). A meta-analysis approach was applied to these datasets as outlined in Figure 2A. As expected, the site (cg20070090) is hypomethylated significantly in the validation datasets. Geometric mean of the cg20070090 was significantly higher for HCC compared to normal samples in each of the five validation datasets.

### Verification of S100A8 methylation by pyrosequencing

We then confirmed the methylation alterations of S100A8 in 52 HCC tissue samples and their adjacent tissues by a highly quantitative method using Pyrosequencing^TM^ technology.

**Figure 3:**
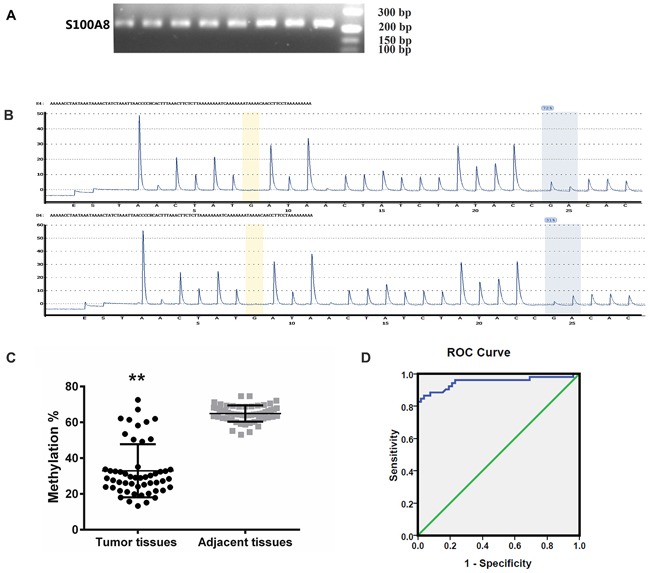
Methylation lever analysis of S100A8 **A.** Methylation-specific PCR of the S100A8 gene. Amplification of bisulfite-treated the target DNA. Methylated PCR products are shown as a 226 bp band. **B.** Pyrosequencing was using to analyse the methylation lever. The methylation density of pyrosequencing is presented in two Pyrogram® charts as the methylation level of the CpG sites analyzed. **C.** Pyrosequencing analysis of the special site of the gene's methylation level in HCC samples and non-tumor samples. Significant differences were observed for S100A8 between tumor tissues and adjacent tissues. Vertical line denotes the grand mean expression value in both groups. **D.** ROC Curve. AUC of S100A8 was calculated to discriminate HCC tissues and the corresponding non-tumor tissues.

For the site of cg20070090, the average DNA methylation levels were significantly decreased(p<0.01) in HCC tissues (32.93%±14.82%) than that in their paired controls (64.91%±4.65%) (Figure 3C).

### ROC curve analysis of S100A8 methylation

To further evaluate the diagnostic significance of S100A8, we constructed a receiver operating characteristic (ROC) curve by plotting the sensitivity versus the specificity (Figure 3D). The area under the ROC curve (AUC) is 0.950 (*p*<0.01). Based on the maximum Youden index discriminating patients with HCC from controls, an optimal cutoff value of 54.025% is obtained and its sensitivity and specificity for predicting HCC is 86.53% and 98.07%, respectively. Our results demonstrated the methylation level of the majority of tumor tissues was less than the cutoff value.

### Association between S100A8 methylation level and clinical, epidemiological, and pathological variables

We analyzed the relationship between the S100A8 methylation level in HCC and various clinical, epidemiological, and pathological variables. However, none of the clinical HCC features was significantly correlated with S100A8 methylation level (Table 2).

**Table 2:** Status of S100A8 methylation in HCC tissues, and their relations to clinical and tumor features

Clinic pathological	Total cases	S100A8 methylation lever (%) [mean ± SD]	p-value
Gender			>0.05
male	47	32.88±15.17	
female	5	33.79±8.73	
Age			>0.05
>66 years old	7	40.40±19.22	
≤66 years old	45	31.77±13.48	
Smoking history			>0.05
Yes	13	36.58±15.99	
No	39	31.17±12.18	
Drinking history			>0.05
Yes	14	34.86±17.49	
no	38	32.22±13.43	
Serum HBV antigen			>0.05
positive	30	30,80±13.23	
negative	22	35.83±16.00	
The AFP in serum			>0.05
>500ng/ml	31	32.06±13.80	
≤500ng/ml	21	34.21±15.80	
Diameter of tumor			>0.05
>5cm	37	34.16±15.39	
≤5cm	15	29.89±12.24	
Portal vein			>0.05
yes	9	27.00±6.73	
no	43	34.17±15.56	
Metastasis			>0.05
yes	5	27.37±12.30	
no	47	33.52±14.80	
Child–Pugh classification			>0.05
A	2	49.91±17.25	
B	39	34.00±15.18	
C	11	24.04±6.63	
Differentiation			>0.05
Moderate–Poor	17	38.80±17.73	
Well-defined	35	30.08±12.63	

### S100A8 methylation level and patient survival

**Figure 4:**
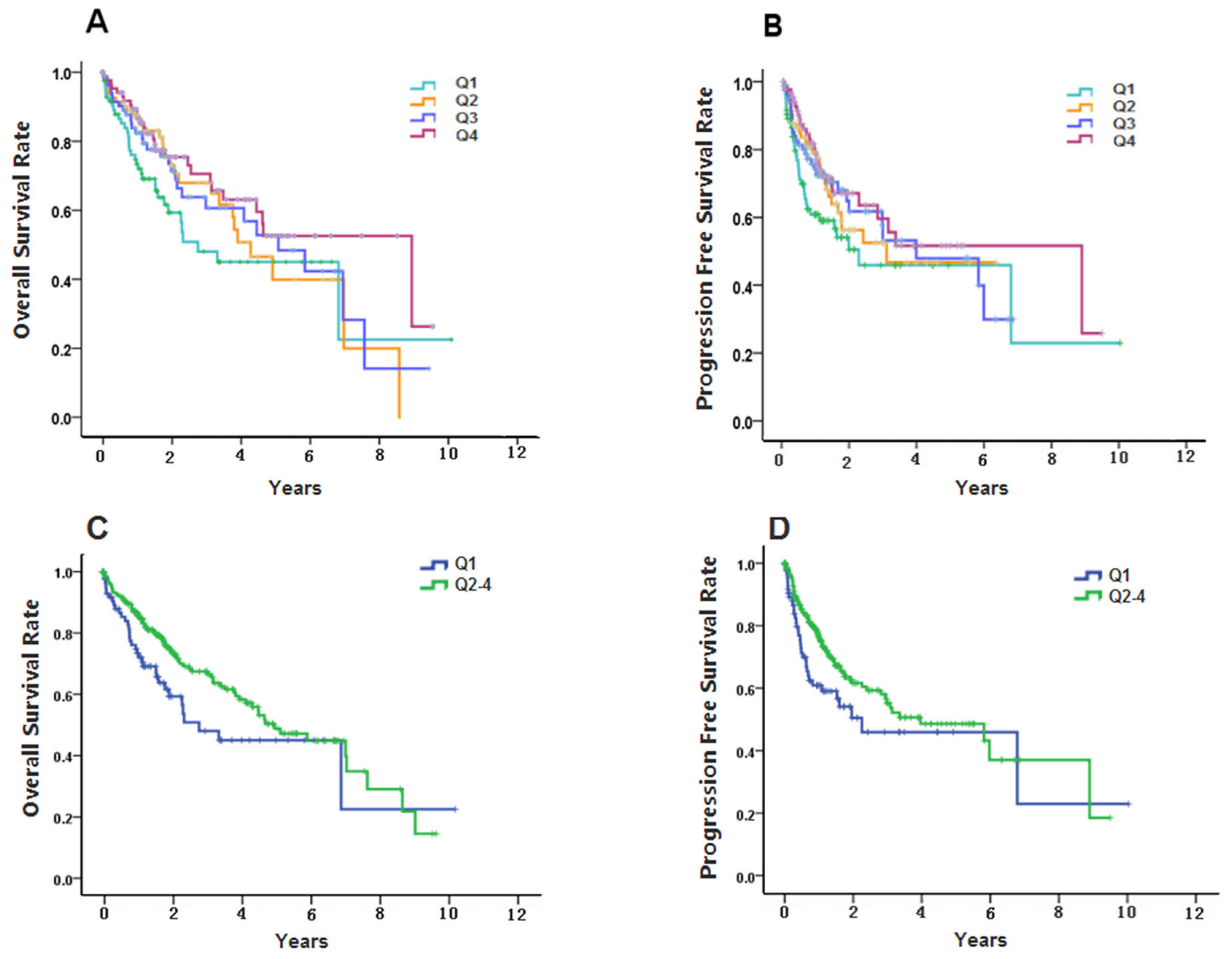
Kaplan–Meier curves of overall survival (OS) and progression-free survival (PFS) of different quartiles of S100A8 methylation level in 345 hepatocellular carcinomas **A.** Kaplan–Meier analysis showed that OS were significantly different among the Q1, Q2, Q3 and Q4 groups (log-rank *p*<0.05). **B.** PFS were significantly different among the Q1, Q2, Q3 and Q4 groups (log-rank *p*<0.05). **C.** OS of Q1 group was significantly longer than the Q2-4 group (log-rank *p*<0.05). **D.** PFS of Q1 group was significantly longer than the Q2-4 group (log-rank *p*<0.05).

We assessed the association of the S100A8 methylation level with survival outcomes. Among 345 patients, 121 of them were dead within a median follow-up of 1.997 years. Based on methylation level of tumor samples, patients were divided into four quartiles (Q1: <25%; Q2: 25%-49%; Q3: 50%-74%; Q4: ≥75%, Supplementary Table S2). The results of Kaplan–Meier analysis showed that OS and PFS were both significantly different among the four groups (Estimate Median OS: Q1:2.753; Q2:4.274; Q3:5.074; Q4:8.926, log-rank *p*<0.05. Estimate Median PFS: Q1:2.266; Q2:3.096; Q3:3.981; Q4:8.926. log-rank, *p*<0.05). Q1 was defined as the ‘low-methylation-level group’ and Q2, Q3, and Q4 were combined into the ‘high-methylation-level-group’. The ‘low-methylation-level group’ was significantly shorter than the ‘high-methylation-level-group’ for OS and PFS (Estimate Median OS time: Q1:2.753; Q2-4:4.907, log-rank *p*<0.05. Estimate Median PFS time Q1:2.266; Q2-4:3.367, log-rank *p*<0.05) (Figure 4, Table 3). Furthermore, according to the results of the multivariate Cox proportional hazards model, OS and PFS were significantly shorter in the ‘low-methylation-level-group’ than in the ‘high-methylation-level group’. The HRs for OS and PFS were 1.709 (95 % CI 1.127–2.591, *p*=0.032) and 1.767 (95 % CI 1.168–2.974, *p*=0.037), respectively.

**Table 3:** Medians (years) for OS and PFS of four groups

group	Estimate	Std. Error	OS		Estimate	Std. Error	PFS	
			95% Confidence Interval				95% Confidence Interval	
			Lower Bound	Upper Bound			Lower Bound	Upper Bound
Q1	2.753	0.949	0.894	4.613	2.266	1.191	0	4.601
Q2	4.274	0.541	3.214	5.334	3.096	.	.	.
Q3	5.074	1.019	3.076	7.072	3.981	1.431	1.176	6.785
Q4	8.926	2.748	3.54	14.312	8.926	3.63	1.812	16.04
Q2-4	4.907	0.684	3.566	6.247	3.981	1.181	1.667	6.295
Overall	4.616	0.711	3.222	6.011	3.367	1.039	1.33	5.404

### Overexpressed S100A8 promotes cell proliferation, migration, invasion, and tumor growth in cultured Huh7 and MHCC-97H liver cancer cell lines

**Figure 5:**
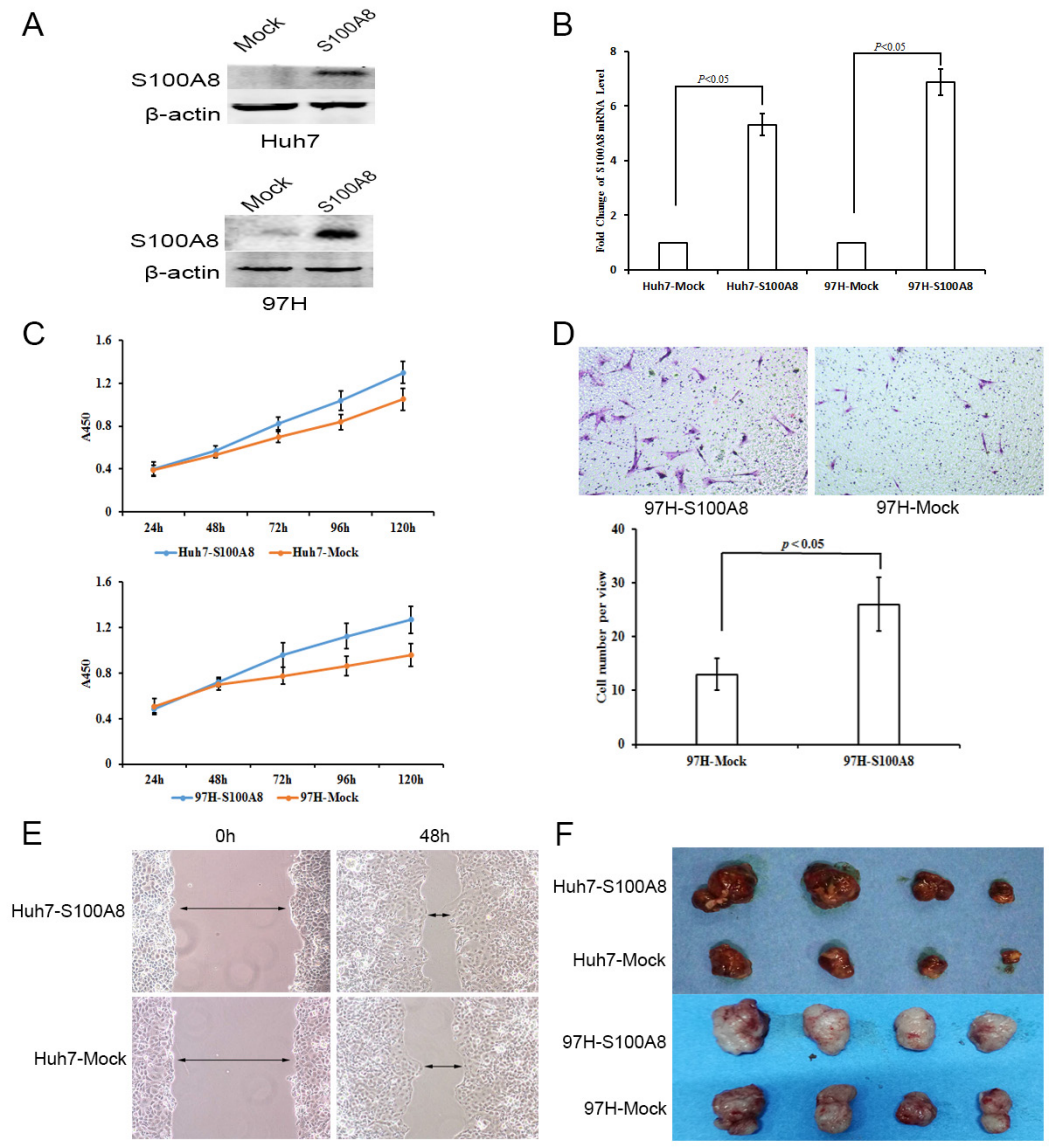
The functional importance of S100A8 in Huh7 and MHCC-97H hepatocellular carcinoma cells **A–B.** Western blot analysis and qPCR were used to detect S100A8 expression in S100A8-transfected Huh7 and MHCC-97H cell lines. **C.** On the basis of CCK8 analysis, S100A8 overexpression in Huh7 and MHCC-97H could inhibit cell proliferation (data are shown as averages ± SD, n = 5). **D.** Scratch-wound assay showed that S100A8 overexpression in Huh7 could promote cell migration. **E.** Overexpression of S100A8 in MHCC-97H could increase cell invasion (data are shown as averages ± SD, n = 3). **F.** Overexpression of S100A8 in Huh7 and MHCC-97H could promote tumor growth in nude mice.

To assess the role of S100A8 in HCC, we established S100A8-transfected Huh7 and MHCC-97H cells by a lentiviral system with puromycin selection. After puromycin selection, S100A8 expression in Huh7 and MHCC-97H cells was measured by western blot analysis and qPCR (Figure 5A and 5B). S100A8 overexpression promoted the cell viability of Huh7 and MHCC-97H compared to that of the empty vector control (Figure 5C). We performed a Scratch-wound assay and cell invasion assay to evaluate the effect of S100A8 on cell migration and cell invasion. The result showed that overexpressed S100A8 significantly promoted the migration of Huh7 cells and invasion of MHCC-97H cells respectively. (Figure 5D and 5E). We also examined the effect of S100A8 on tumorigenicity in vivo using subcutaneously tumor model in nude mice. Compared to the control group, S100A8 overexpression resulted in significant promotion of tumor growth (Figure 5F)

## DISCUSSION

Methylation alterations of tumor-associated genes are frequently observed in the development of cancer and may occur at different stages of HCC [33–37]. We used the genome-wide methylation array to screen the HCC-specific methylation alterations, and found methylation level of the site (cg2007009) in S100A8 decreased significantly in HCC. Our result was confirmed by the meta-analysis of 5 publicly available methylation datasets, which greatly improved the robustness and credibility of our finding. Furthermore, we assessed the value of S100A8 methylation in HCC diagnosis, AUC was 0.950, which is higher than AFP(0.902), vascular endothelial growth factor (VEGF) (0.841) and serum leptin (Lep) (0.712) [38]. Although it still needs to be verified by a large amount of data and clinical experiments, our data certainly supported a potential role for S100A8 hypomethylation as a diagnostic HCC biomarker.

As is known to all, the prognosis of HCC is related to the patient's age, gender, liver function, tumor metastasis, pathological changes, the level of AFP, the activity of immune cells, the number of platelets, and so on [39–41]. However, there are few reports about the relationship between the methylation level of S100A8 and the prognosis of liver cancer. After analyzing the samples with clinic information in TCGA database, we found that the average OS and PFS of the HCC group with a relatively higher degree of methylation had a longer survival time than that of lower degree group. The patients with lower S100A8 methylation level showed poorer PFS and OS rates compared with S100A8 high-level group. This may indicate that one site of S100A8 methylation level can be used as a molecular biomarker for the prognosis of HCC. However, due to the lack of survival time in our data, a large well designed cohort is warranted to get a more precisely prediction result in China.

S100A8 is involved in the regulation of a wide range of cellular processes, such as cell proliferation, the dynamics of cytoskeleton components, differentiation, Ca^2+^ homeostasis, and apoptosis [19, 20]. Interestingly, S100A8 has been found to be upregulated incolorectal carcinoma, lung cancer, pancreatic cancer, breast cancer and thyroid carcinoma [23–30]. S100A8 ablation in mice can decrease cell proliferation, leading to significant reduction of tumor size [42]. S100A8 treatment can increase the viability and migration of colorectal carcinoma cells [43].We performed a series of experiments to evaluate the role of S100A8 in HCC. The results showed that the S100A8 overexpression in Huh7 and MHCC-97H cells induced marked increase in cell proliferation, migration, invasion, and tumor growth. These results suggested that S100A8 might be a tumor promoter in HCC cells.

In summary, our data demonstrated that S100A8 were frequently hypermethylated in non-tumor tissues but hypomethylated in HCC tissues. The AUC is higher than other general biomarkers of HCC. Hypomethylation of S100A8 was associated with both a shortened PFS and OS. The results of this study indicate that detection of S100A8 methylation level would be helpful to diagnose HCC and predict HCC patient prognosis. Methylation of S100A8 is a promising diagnosis and prognostic marker in HCC. Moreover, we demonstrated that S100A8 was probably a tumor promoter of HCC.

## MATERIALS AND METHODS

### Cell lines

HCC cell line Huh7 was purchased from the Chinese Academy of Sciences, Shanghai, China. HCC cell line MHCC-97H was established at the Liver Cancer Institute, Zhongshan Hospital, Fudan University. The cells were maintained in DMEM supplemented with 10% fetal calf serum. The cells were incubated at 37°C in a humidified chamber containing 5% CO_2_.

### Patients and tissue samples

HCC tumor tissue samples and paired adjacent normal tissue samples were obtained by hepatectomy from the First Affiliated Hospital of Guangxi Medical University, following written informed consent and approved by the ethics committee. All tumors were classified according to the International Union Against Cancer tumor–node–metastasis classification system and World Health Organization criteria. Clinic pathologic data for parameters such as patient's age, gender, original tumor size, infection of HBV, serum level of AFP, portal vein tumor embolus, and degree of differentiation were collected. All tissue samples were stored in liquid nitrogen.

### DNA isolation and bisulfite conversion

DNA was exacted by a QIAamp DNA Mini Kit (Germantown, MD, USA). Genomic DNA was then bisulfite-converted with an EpiTect Bisulfite Kit (QIAGEN) according to manufacturer's protocol.

### Methylation microarrays

Bisulfite-converted genomic DNA was analyzed by The Infinium Methylation-27 Assay. Chip processes were performed by using reagents provided in the kits and following manufacturer's manual. Data were extracted and summarized using GenomeStudio software. Arrays that did not pass quality control in terms of b-distriutions and expected p-values across the arrays were removed. Methylation scores represented as β values were generated for each site using Illumina Genome Studio Methylation module v1.8 (Illumina Inc., USA) and were computed based on the ratio of methylated to methylated plus ummethylated signal outputs. Control panel in the BeadStudio analytical software showed excellent intensity for staining, clear clustering for the hybridization probes, good target removal intensity and satisfactory bisulfite conversion [44].

### Data collection from public databases

Four methylation datasets (accession numbers GSE54503, GSE44909, GSE37988, GSE57956) generated by the Illumina Infinium HumanMethylation27K or 450K platforms were downloaded from the NCBI GEO database. Background correction was performed by Illumina GenomeStudio software, which were declared by the data contributor in GEO database. And for GSE54503 dataset, we also performed the beta-mixture quantile normalization (BMIQ) [45]. Furthermore, we downloaded DNA methylation data with clinical data of HCC from The Cancer Genome Atlas (TCGA). Data processing was performed using RnBeads software. We thus use the same background correction-method with GenomeStudio software and BMIQ method were used for the normalization process. After excluding aberrant samples by probe quality control, Principal Components Analysis (PCA) and clustering, 345 patients' data including 345 tumor tissues and 50 adjacent normal tissues were obtained. Totally, 246 HCC samples and their matched adjacent normal tissues from five datasets were eligible for meta-analysis. Only TCGA dataset was used for survival analyses.

### Meta-analysis

A meta-analysis approach was applied to the normalized data. It combines effect sizes from each dataset into a meta-effect size to estimate the amount of change in expression across all datasets. For the site of the gene in each dataset, an effect size was computed using Hedges' adjusted. The study-specific effect sizes were combined to obtain the pooled effect size and its standard error using the random effects inverse-variance technique. The Z-statistic was computed as a ratio of the pooled effect size to its standard error for the gene, and the result was compared to a standard normal distribution to obtain a nominal *p*-value.

### Pyrosequencing analysis of DNA methylation

The primers were designed using Pyrosequencing^TM^ Assay Design Software Version 1.0; where one of the primers was biotin-labeled. Primer sequences were as follows: Sense primer biotin-5′-GGAA GGTGTTGGAGGATATT-3′, antisense primer 5′-CCT CTCAACCCTACATATCTCTTATCA-3′, sequencing primer 5′-ATCTCTTATCAACTATCTTTCA-3′. The Platinum PCR SurperMix High Fidelity (Invitrogen, Carlsbad, CA) was used to prepare the PCR reaction solution. After the biotinylated strand was captured on streptavidin-coated beads (Amersham Bioscience, Uppsala, Sweden) and incubated with sequencing primers, the specific PCR products were then subjected to quantitative pyrosequencing analysis using a Pyrosequencing™ PyroMark MD system following the protocol provided by the manufacturer. The sequencing results were analyzed using the PyroMark Q96 software (Qiagen).

### Construction of S100A8 lentiviral vectors

To investigate the effect of S100A8 overexpression on HCC cell lines, S100A8 (NM_002964.4) lentiviral vector with a puromycin selection marker was constructed (Genecopoeia Co. Ltd., Guangzhou, China), and empty vectors were used as controls.

### Western blot analysis

Equal amounts of total proteins (20μg) were separated by 10% SDS-PAGE and transferred onto PVDF membrane using a Bio-Rad SemiDry apparatus. The membrane was blocked by 5% milk or 2% BSA at room temperature for 1 h. Then, the membrane was incubated with specific primary antibody with suitable dilution at 4°C overnight. After 3 times of 10 min washing by TBST, the membrane was further incubated with HRP-conjugated secondary antibodies (Bio-Rad) at room temperature for 1 h, and then washed again by TBST for 3 times of 10 min. ECL Western Blotting Detection Reagents (Bio-Rad) and ChemiDoc XRS^+^ system (Bio-Rad) were used to visualize the bands on membrane.

### Cell proliferation assays

Huh7 cells and MHCC-97H cells (1000 cells/well) were dispensed in 100 μL aliquots into a 96-well plate. At the indicated time points, the 2-(4-indophenyl)-3-(4-nitrophenyl)-5-(2,4-disulphophenyl)-2H-tetrazolium monosodium salt (CCK8, Cell Counting kit) was added to the cells for 1h, and then the plate was read using an enzyme-linked immunosorbent assay plate reader at 450 nm.

### Scratch-wound assay

Seed cells into 24-well tissue culture plate at a density that after 24 h of growth, they should reach 70-80% confluence as a monolayer. Gently and slowly scratch the monolayer with a new 200μl pipette tip across the center of the well. Scratch a straight line in one direction. Scratch another straight line perpendicular to the first line to create a cross in each well. After 48 hours incubation at 37 °C, take photos on a microscope. The gap distance can be quantitatively evaluated using Photoshop. Each experimental group should be repeated 3 times.

### Cell invasion Assay

For in vitro invasion assay, transwells coated with Matrigel (Millipore) were utilized according to manufacturer's instructions. 5 × 10^4^ cells in 100 μl serum-free DMEM were seeded in the upper chamber of a transwell and 500 μl medium contain 15% FBS was added to the lower chamber. After 30 hours incubation at 37 °C, invaded cells were fixed with 20% methanol and stained with Hematoxylin Staining Solution. Cells adhering to the lower side of the inserts were counted and imaged through microscope. Five random microscopic fields were counted per well for each group, and the experiments were repeated at least three times independently.

### Tumor formation assay

Male BALB/C nude mice (5-6 week old) were obtained from Shanghai Institute of Materia Medica (Chinese Academy of Sciences, Shanghai, China). The in vivo experiments were carried out strictly in accordance with a protocol approved by the Guangxi Medical University Experimental Animal Care Committee. 1×10^7^ cells were injected subcutaneously into the upper left flank region of nude mice. The mice were sacrificed at the 28th day after cell injection.

### Statistical analysis

Statistical analyses were performed using the SPSS 16.0 software package. Data were expressed as mean ± SD. One-way ANOVA was employed to determine the differences among different groups. Pearson partial correlation analysis was used to analyze the correlation of multiple variables. Receiver operating characteristic (ROC) curves were used to determine the diagnostic value of the markers. The best cut-off value for each protein was defined as the point with maximum Youden index (sensitivity+ specificity −1) on the ROC curve. Survival curves were generated using the Kaplan-Meier method, and comparisons between the curves were made using the log-rank test. Patients were divided into four quartiles based on methylation level of tumor sample. Overall survival (OS) and progression-free survival (PFS) were compared between the quartiles. OS was taken from “days to death” for deceased patients and “days_to_last_followup” for patients reported living. PFS was taken from patients with reported “days to new tumor event”, and PFS equal to OS for blank or “NA” entries. Multivariate survival analyses were performed with the Cox proportional hazards model. The reference groups were ‘high-methylation-level group’. *p*< 0.05 was considered statistically significant.

## SUPPLEMENTARY MATERIALS TABLES




