# Supplementary material for: Methylation of S100A8 is a promising diagnosis and prognostic marker in hepatocellular carcinoma
Source: Oncotarget. 2016 Jul 23;7(35):56798–810. doi: 10.18632/oncotarget.10792 (PMC5302953; doi:10.18632/oncotarget.10792)
Supplement: Supplementary file 1 [file oncotarget-07-56798-s001.pdf]

Methylation of S100A8 is a promising diagnosis and prognostic marker in hepatocellular carcinoma

SUPPLEMENTARY TABLES

Supplementary Table 1: Information of GEO studies for Meta-analysis

GEO accession / Source	Authors	Institute	Tumor samples	Normal samples	Total samples
GSE54503	Shen J *et al*.	Columbia University, USA	66	66	132
GSE44909	Revill K *et al*.	Cold Spring Harbor Laboratory, USA	12	12	24
GSE37988	Shen J *et al*.	Columbia University, USA	62	62	124
GSE57956	Mah W *et al*.	National Cancer Centre, Singapore	56	56	112
TCGA	NCI and NHGRI	The National Cancer Institute (NCI) and National Human Genome Research Institute (NHGRI), USA	50	50	100
Total		5 cohorts	246	246	492

Supplementary Table 2: Characteristics of TCGA data of four groups

See Supplementary File 1
