# Supplementary material for: Methylation of S100A8 is a promising diagnosis and prognostic marker in hepatocellular carcinoma
Source: Oncotarget. 2016 Jul 23;7(35):56798–810. doi: 10.18632/oncotarget.10792 (PMC5302953; doi:10.18632/oncotarget.10792)
Supplement: Supplementary file 2 [file oncotarget-07-56798-s002.docx]

**Supplemental Table 2. Characteristics of TCGA data of four groups.**

| Clinic pathological | Total | Q1 | Q2 | Q3 | Q4 |
| --- | --- | --- | --- | --- | --- |
| Gender |  |  |  |  |  |
| male | 236 | 60 | 62 | 60 | 54 |
| female | 109 | 27 | 24 | 27 | 33 |
| Age |  |  |  |  |  |
| (mean ± sd) | 89.91+12.90 | 61.28±11.99 | 63.69±11.14 | 59.10±12.46 | 55.63±14.35 |
| Smoking history |  |  |  |  |  |
| Yes | 13 | 2 | 4 | 3 | 4 |
| No | 332 | 85 | 81 | 84 | 83 |
| Drinking history |  |  |  |  |  |
| Yes | 112 | 32 | 32 | 28 | 20 |
| no | 233 | 55 | 54 | 59 | 67 |
| Hepatitis |  |  |  |  |  |
| positive | 259 | 69 | 60 | 65 | 65 |
| negative | 86 | 18 | 26 | 22 | 22 |
| Race |  |  |  |  |  |
| Asian | 152 | 47 | 29 | 37 | 39 |
| Blank OR African American | 16 | 4 | 6 | 0 | 6 |
| White | 166 | 35 | 46 | 45 | 40 |
| Family |  |  |  |  |  |
| Yes | 97 | 20 | 31 | 25 | 30 |
| No | 153 | 67 | 55 | 62 | 57 |
| Unknown | 46 | 15 | 15 | 10 | 6 |
| recurrence |  |  |  |  |  |
| Yes | 162 | 46 | 40 | 37 | 39 |
| No | 183 | 41 | 45 | 49 | 48 |
| Topography |  |  |  |  |  |
| T1 | 171 | 37 | 45 | 38 | 51 |
| T2 | 85 | 25 | 21 | 23 | 16 |
| T3 | 74 | 21 | 15 | 19 | 19 |
| T4 | 1 | 0 | 0 | 0 | 1 |
| Lymph Node |  |  |  |  |  |
| N0 | 236 | 65 | 53 | 57 | 61 |
| N1 | 3 | 1 | 1 | 1 | 0 |
| NX | 105 | 21 | 31 | 28 | 25 |
| Metastasis |  |  |  |  |  |
| M0 | 250 | 67 | 59 | 61 | 63 |
| M1 | 4 | 0 | 0 | 0 | 3 |
| MX | 91 | 19 | 26 | 25 | 21 |
| Stage |  |  |  |  |  |
| Ⅰ | 161 | 35 | 43 | 35 | 48 |
| Ⅱ | 78 | 24 | 20 | 21 | 14 |
| Ⅲ | 79 | 24 | 31 | 41 | 17 |
| Ⅳ | 6 | 1 | 0 | 2 | 3 |
